# Firearms-related skeletal muscle trauma: pathophysiology and novel approaches for regeneration

**DOI:** 10.1038/s41536-021-00127-1

**Published:** 2021-03-26

**Authors:** Anselmo Moriscot, Elen H. Miyabara, Bruno Langeani, Antonio Belli, Stuart Egginton, T. Scott Bowen

**Affiliations:** 1 Department of Anatomy, Institute of Biomedical Sciences, University of São Paulo, São Paulo, Brazil; 2 Sou da Paz Institute, São Paulo, Brazil; 3 NIHR Surgical Reconstruction and Microbiology Research Centre, University of Birmingham, Birmingham, UK; 4 School of Biomedical Sciences, Faculty of Biological Sciences, University of Leeds, Leeds, UK

**Keywords:** Translational research, Mechanisms of disease, Ubiquitin ligases, Stem-cell research

## Abstract

One major cause of traumatic injury is firearm-related wounds (i.e., ballistic trauma), common in both civilian and military populations, which is increasing in prevalence and has serious long-term health and socioeconomic consequences worldwide. Common primary injuries of ballistic trauma include soft-tissue damage and loss, haemorrhage, bone fracture, and pain. The majority of injuries are of musculoskeletal origin and located in the extremities, such that skeletal muscle offers a major therapeutic target to aid recovery and return to normal daily activities. However, the underlying pathophysiology of skeletal muscle ballistic trauma remains poorly understood, with limited evidence-based treatment options. As such, this review will address the topic of firearm-related skeletal muscle injury and regeneration. We first introduce trauma ballistics and the immediate injury of skeletal muscle, followed by detailed coverage of the underlying biological mechanisms involved in regulating skeletal muscle dysfunction following injury, with a specific focus on the processes of muscle regeneration, muscle wasting and vascular impairments. Finally, we evaluate novel approaches for minimising muscle damage and enhancing muscle regeneration after ballistic trauma, which may have important relevance for primary care in victims of violence.

## Introduction

Traumatic injuries have a major impact on society, contributing to around 10% of global disease burden^1^. One major cause of traumatic injuries is firearm-related wounds (i.e., ballistic trauma), which are common occurrences in both civilian and military populations. In addition to acute incapacity, ballistic trauma has serious long-term health and economic consequences. For example, in the USA the incidence of firearm injuries surpasses 100,000 per year with around 30,000 of these cases fatal, yet clinical management and treatment of such wounds remains suboptimal^2^. Furthermore in Brazil, a country with one of the world’s highest rate of firearm-related homicides^3^, it has been estimated that firearm injuries alone cost an estimated 88 million US dollars each year, and when extended to indirect costs (e.g., loss to workforce, policing etc) has been estimated to be around 10 billion US dollars per year (or 0.5% of GDP)^4^. Alarmingly, the prevalence of firearm injuries are reported to be increasing, even in developed countries such as the UK and USA^5^. Indeed, firearm incidents are now under more scrutiny than ever: nearly half of all global homicides can be attributed to firearms^3^ while there has been a sharp rise over recent years in lone terrorist gun attacks across developed nations such as Canada, USA, Norway, and the UK, which has attracted huge media attention. In fact, most violent deaths that include use of firearms do not occur in direct conflict zones but rather among civilians, which now accounts for 74% of total violent deaths worldwide^3^. In relation to modern warfare, such as recent direct conflicts in Afghanistan and Iraq, evidence shows firearm wounds and explosive blasts account for almost all of the suffered injuries^6^.

Following ballistic trauma, the most common primary consequences include soft-tissue damage, volumetric muscle loss (VML), haemorrhage, bone fractures, and pain^7^. The major site of firearm wounds are of musculoskeletal origin in the extremities in both military^6^ and civilian populations^8^. Indeed, injuries promoted by firearm incidents that impact skeletal muscle induce severe disability, extended hospitalisations, and overall a poor quality of life^9^. Collectively, therefore, current evidence suggests skeletal muscle should be a major therapeutic target following firearm-related injuries, and improving its regeneration will promote recovery and quality of life in afflicted patients. In this review, we will address a global issue that has major socioeconomic impact, with a specific focus on skeletal muscle damage and regeneration. We will start with a brief historical perspective of the early medical treatments developed for firearm-related wounds. After this, we will review basic trauma ballistics and the immediate damage they inflict on skeletal muscle, followed by what modern clinical management procedures are in place to deal with such injuries. We then provide an in-depth analysis of the underlying biological mechanisms responsible for regulating skeletal muscle dysfunction following traumatic injury, including the interlinked processes of muscle regeneration, muscle wasting and vascular impairments. Finally, we discuss novel cutting-edge approaches that may soon help alleviate muscle dysfunction and promote muscle regeneration in even the most severely impacted patients with firearm injuries.

## Historical perspective

We are naturally attracted to recent advances in our own field of research, and often miss important insights from earlier, less time-pressured studies. As Theodor Billroth (1826–1894), a pioneering Austrian surgeon, said ‘Only the man who is familiar with the art and science of the past is competent to aid in its progress in the future’. He made observations largely in accord with modern descriptions of sprouting angiogenesis, where strands of cells arise from existing vessels and extracellular canalisation takes place to form a patent lumen^10^. Treatment of penetrating wounds from projectile fragments was a necessity from the earliest times, essentially tending collateral damage of ballistic bombardment. However, the advent of weapons targeting individuals brought new challenges that benefitted from a more holistic approach in patient management. The first, very brief reference to gunshot wounds was by Billroth (1856)^10^, and the oldest work on surgery with a short chapter on gunshot wounds appeared not long after by Hieronymus Brunschwig (1497)^11^ It was common to illustrate the injuries that one may have to treat by means of a stylised figure with examples of their location and/or form. At this time, the ‘wound man’ of the barber-surgeon takes the place of the ‘zodiac figure’ previously referred to by physicians. However, no gunshot wounds are apparent, likely reflecting both the rarity and lack of knowledge regarding effective treatment at that time. The main aim was to remove poison (gunpowder residue and battlefield contaminants) by enlarging the wound and application of herbal poultices using warm oil to clean, adding swelling (inflammatory) packs, followed by stimulating ointments. Although early treatment was orientated towards binding open wounds and allowing self-healing, advances in anatomical knowledge gradually led to a better understanding that different components of damaged tissue required attention. For example, drawings by the Belgian anatomist Andreas Vesalius (1514–1564) highlighted the similarities in arborisation of the vascular and neural networks on a gross scale during dissection of human cadavers, consistent with similar observations outlined by the great Leonardo da Vinci (1452–1519).

The most significant leap in practice appears to be due to the efforts of one man, John Hunter (1728–1792), credited with establishing rigorous surgical practice in both Britain and Germany. He was appointed senior surgeon for a British army ‘expedition’ to Portugal (1761), and surgeon general of the army (1790). Based on his experience in the field, he published his monograph ‘A treatise on the blood, inflammation and gunshot wounds’^12^, the first on the subject, which was still referred to in the clinical literature nearly a century later. His observations were on a wide canvas, including the context that ‘Fire arms and spirits are the first of our refinements that are adopted in uncivilised countries’. Unusually, his attempts to improve battlefield care was based not only on documented experience, but on experimental observations using studies on mammals, birds, fishes and insects. Hunter’s greatest contributions towards effective treatment involved describing: (1) the difference between gunshot wounds and common wounds; (2) the difference between entry and exit wounds; (3) the effects arising from different musket ball velocity; and (4) that ‘wound blush’ is diagnostic for closure/wound repair (i.e., the extent of vascularisation is proportional to healing potential—a principle re-discovered in the twentieth century when tissue grafts became a surgical routine).

Hunter also explored the importance of vascular growth in tissue remodelling in other ways, describing carotid artery dilation of stags during the rutting season to sustain antler growth, and introduced the concept that was later to be called angiogenesis in 1787 to describe the growth of blood vessels in reindeer antlers as a result of long exposure to cold. Although often attributed to Hunter, the term ‘angiogenesis’ was probably first used much later^13^. In relation to wound healing, the role of angiogenesis was first described by Travers (1843)^14^, showing that new blood vessels grow from pre-existing vessels, later erythrocytes are seen with oscillatory behaviour for a few hours, then a normal circulation is established (consistent with modern intravital microscopic observations). Further studies by the Clarks (a husband and wife team from 1918–1940^15^) demonstrated that a major formative force during development was rate of flow (we now know this to be shear stress mechanotransduction), rising from 0 to 4 mm s^−1^ in 30 days: differentiation was seen in vessels with high flow, while those with low flow regressed. Subsequently, Cliff^16^ and Schoefl^17^ used more modern experimental techniques, combining light and electron microscopy, when studying wound healing in skin and muscle to reveal novel structural developments. This helped lay the foundations for numerous molecular and bioengineering studies currently exploring tissue regenerative potential. The first successful muscle graft involved a transplanted part of a previously denervated rat gastrocnemius, with histological proof of survival, by Studitsky & Bosova in 1960^18^. In 1971 Thompson published preliminary experimental and clinical results, and a number of other papers followed from the pioneering labs of Carlson, Gutmann, Hakelius etc. addressing diverse conditions such as correction of facial paralysis and survival of minced muscle tissue. With the introduction of modern infrastructure, dedicated medical care trauma centres are now the norm across the world for treatment and rehabilitation of firearm-related injuries^19^. However, it would be wise to remember that many of our modern clinical advances in this area were made possible by the early courageous work of the first pioneers investigating gunshot wounds on the battlefield.

## Wound ballistics and clinical management

**Fig. 1:**
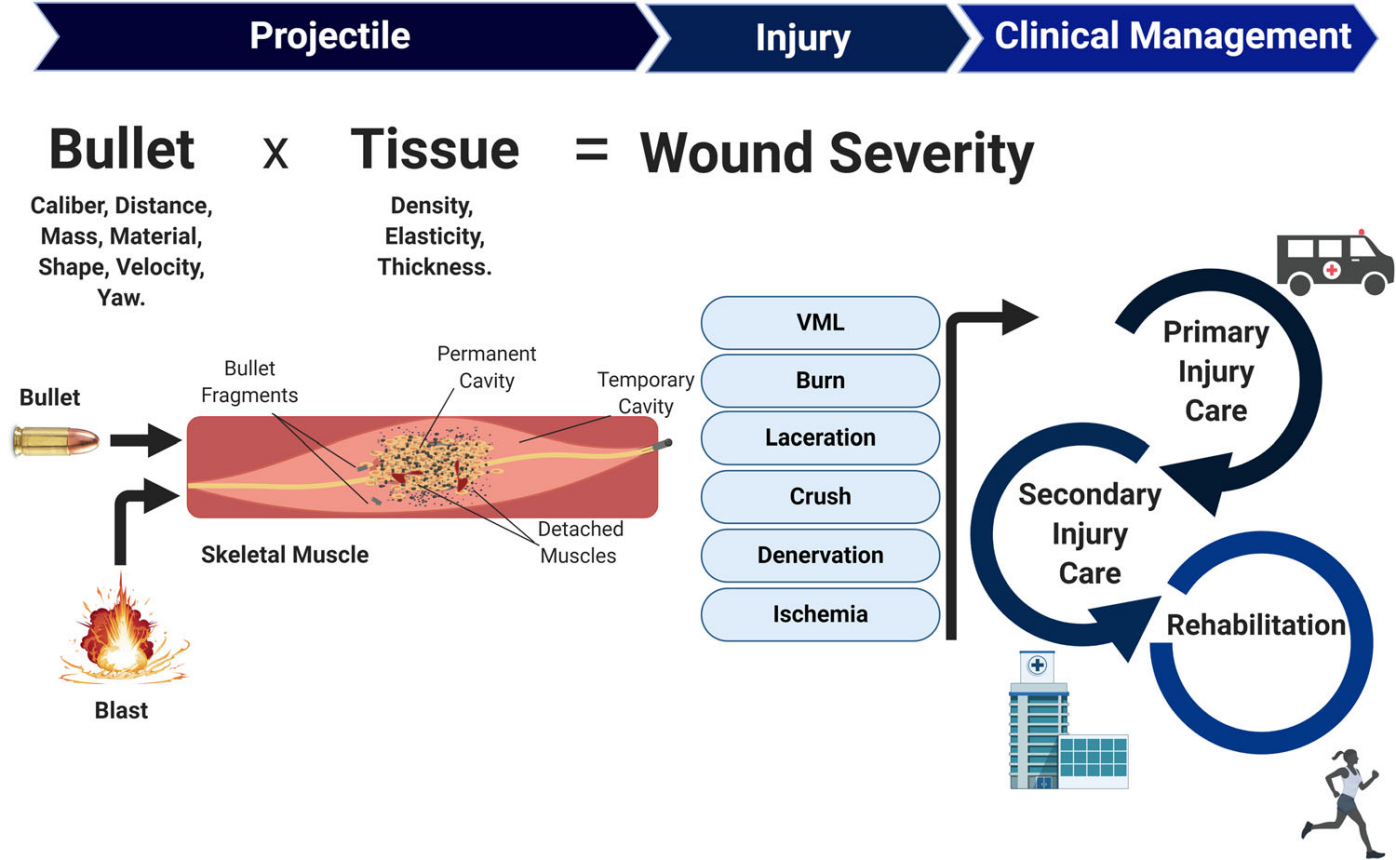
Summary of the key considerations involved regarding firearm-related muscle trauma and its clinical management. The properties of the projectile (i.e., bullet or explosive blast fragments) and muscle tissue impacted interact to determine the severity of the wound, which is dependent upon permanent and temporary cavitation as well as bullet fragmentation. The type of injury induced may range from very severe (such as volumetric muscle loss; VML) to mild (such as a minor laceration). The speed of the clinical response and management play a hugely important role for determining patient outcomes and functional recovery, which includes primary and secondary treatment alongside long-term rehabilitation.

Wound ballistics refers to the study of projectiles that penetrate the human body with particular reference to the injury inflicted, which can include both bullet and blast/explosive traumas^20^. Impact velocities with lower limits of ~150 feet s^−1^ are known to penetrate the skin to cause significant injury^9^. The main mechanisms underlying penetrating ballistic tissue wounds associated with bullets include: (1) permanent cavitation, whereby tissue is destroyed by projectile compression and shearing that leaves a projectile trail; and (2) temporary cavitation, caused by tissue distention due to high-energy pressure vibrations from the projectile’s trail (Fig. 1). Other issues, such as localised frictional heat stress, may exacerbate these primary insults. The extent of the firearm wound (i.e., depth and area damaged) is highly dependent upon projectile-tissue characteristics, whereby both the projectile (e.g., velocity, mass, shape, calibre, material, yawing and impact distance) and tissue impacted (e.g., density, elasticity, and thickness) interact to define the full extent and nature of injury^21^ (Fig. 1). In general, skeletal muscle is suggested to be more sensitive to permanent cavitation, with temporary cavitation thought to induce less damage (unless the vasculature is disrupted) due to skeletal muscle’s inherent elasticity^21^.

A common misconception is that higher projectile velocities (e.g., >600 m s^−1^) always induce greater wound severity compared to low-velocity projectiles. However, extensive wounds can also be caused by bullet fragmentation that expand wound area despite low velocity. For example, a commonly used firearm such as the shotgun is technically defined as low velocity, yet this device inflicts devastating wounds when fired at close range due to high-energy transfer^21,22^. Indeed, the amount of energy transferred by the projectile is a major factor contributing to wound severity, typically subdivided into low- or high-energy injuries. Explosive blasts in particular, which are often suffered during military warfare, are key examples of high-energy injuries that cause complex muscle trauma by inflicting both superficial and deep regional damage. Blast wounds are caused by the primary ‘explosive wave’ (which can lead to immediate air-related organ damage and fractures; *non-penetrating injuries*), the secondary ‘blast wave’ (i.e., flying bomb and other fragments; *penetrating injuries*), and the tertiary wave causing body displacement (i.e., the blast wind flinging the body to cause *crush injuries*)^23^. Explosive blasts often encapsulate the most devastating physical injuries due to multiple effects that result in penetrating, crushing, thermal, and infectious wounds. In regards to skeletal muscle impacted by a projectile or blast injury, damage is inflicted via various routes with laceration, contusion/crush injury, denervation (i.e., neural deficits), haemorrhage/ischaemia (i.e., vascular impediments) burns, and VML in particular of concern (Fig. 1). Both primary firearm or blast wounds can be aggravated by secondary trauma that further complicates severity of the injury, including development of infection/sepsis (caused in some cases by contamination with bullet/shotgun wadding, or other debris collected from clothing or skin), surgical debridement of damaged tissue (optimally performed within 6–8 h of trauma), and/or excessive physical movement^9^.

The clinical management following ballistic trauma can be generally categorised into distinct stages, including: (1) primary care (on-site treatment and transport time); (2) secondary care (usually hospital-based interventions); and (3) rehabilitation (location dependent on severity) (Fig. 1). Organised trauma care systems have been significantly strengthened worldwide over recent decades to substantially reduce mortality rates^19^. One key factor that is strongly predictive of outcome following major trauma injuries, whether military or civilian, is the time taken to receive medical attention^24,25^, underlying the ‘golden hour’ policy mandating rapid trauma care from call to arrival. Following primary treatment at the site of injury, patients are then rapidly transported to organised trauma care centres (if available) for optimised treatment, where specialist teams and equipment are available^19^. Secondary care will then involve any further clinical interventions, and in the case of ballistic trauma often include surgical debridement and tissue reconstruction. The recovery process then follows, where targeted rehabilitation strategies such as physical therapy are commonly employed to aid recovery (Fig. 1). Treatment and survival have improved with modern day trauma care systems^19^, with in-hospital, 30 and 60 day mortality rates reduced by up to 30% compared to standard care^2^. However, in regards to firearm wounds we still have a poor understanding of the underlying pathophysiological mechanisms involved and limited strategies to help regenerate damaged muscles, thus severely impeding rapid recovery of many patients.

## Pathophysiology of skeletal muscle trauma

**Fig. 2:**
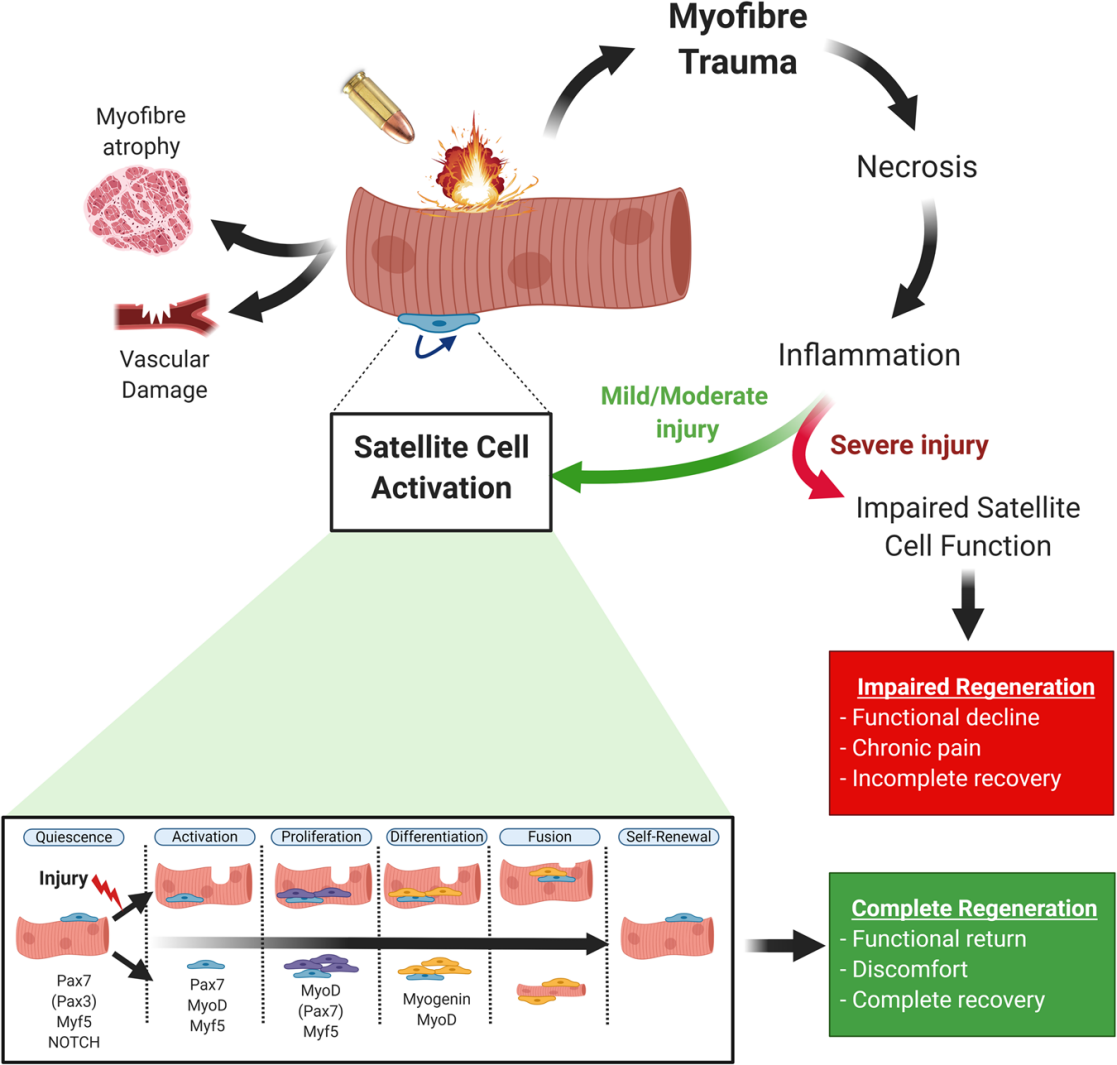
Overview of the mechanisms involved in the muscle injury-regeneration response following projectile trauma. Ballistic trauma would induce immediate myofiber trauma, alongside secondary atrophy and vascular damage. Myofibre trauma initiates a chain of events, including necrosis and inflammation, which then activates satellite cells to aid muscle regeneration. Satellite cells progress through a well-established cycle of events, starting from activation and ending with fusion, which is tightly controlled by numerous transcription factors (bottom panel; e.g., Pax7, MyoD)^73^. Patients that suffer mild to moderate muscle damage have a high chance of complete recovery, due to the muscle regeneration process proceeding as normal. In contrast, patients with a severe muscle injury will show impaired recovery, due to extensive loss of muscle tissue and impaired satellite cell function that inhibits muscle regeneration with chronic pain and fibrosis. Secondary atrophy and vascular damage can also contribute to impaired muscle regeneration via inhibiting normal satellite cell function. Bottom panel is adapted from Baghdadi and Tajbakhsh^73^.

Given experimental (lack of suitable animal models) and ethical (need to avoid deliberate suffering) limitations, our understanding of the pathophysiology of skeletal muscle trauma and regeneration in humans following ballistic wounds remains far from complete, with much of our knowledge gleaned from uncontrolled settings involving civilian or military injuries. Indeed, most of our mechanistic understanding related to projectile muscle trauma and regeneration have come from indirect preclinical trauma models rather than direct ballistic wounds. Alternative approaches generally include muscle wounds induced by either direct physical damage (e.g., VML, contusion, laceration, ischaemia, denervation, freeze) or more indirect chemical damage (i.e., myotoxic agents, barium chloride, irradiation)^26^, which have different temporal and spatial consequences for both muscle damage and regeneration that should be carefully considered (as reviewed elsewhere^27,28^). Other approaches specific to projectile trauma have also used soap or gelatin produced with similar physical properties to human muscle tissue in order to investigate properties of a projectile impact^20,21^. One key aspect to improving muscle injury and regeneration after ballistic trauma is by first understanding how the muscle immediately responds to injury or trauma, which we will now briefly review. In particular, the ability of the muscle to regenerate is highly dependent upon a population of muscle-specific progenitor cells termed satellite cells^29^. These are muscle stem cells juxtaposed to myofibers sheathed under the basal lamina, which typically reside in a quiescent state but are capable of regenerating damaged muscle fibres upon stimuli inducing their activation^29,30^ (Fig. 2).

### Acute response to myofiber trauma

Immediately following a muscle insult (e.g., bullet), a necrosis of damaged myofibers occurs, which is accompanied by an increase in intracellular calcium concentration with consequent proteolysis of the damaged tissue^30,31^. Subsequently, key inflammatory cells (neutrophils) are the first cells recruited to damaged tissue (within 6 h after muscle injury)^30,32^. Next, pro-inflammatory macrophages (also called M1 macrophages) infiltrate the damaged tissue, which peak around 24 h after injury and are responsible for phagocytosis and release of pro-inflammatory cytokines such as interleukin (IL)-1 and IL-4 that promote satellite cell proliferation^31,33,34^. Anti-inflammatory macrophages are most prevalent in the injured region between 2 to 4 days post-damage and secrete anti-inflammatory cytokines that facilitate irreversible differentiation of satellite cell-derived myoblasts, and thus subsequent fusion and myofibre regeneration^33,34^. Signalling of M2 macrophages is key for triggering the permanent differentiation of satellite cell-derived myoblasts, which fuse (either with each other or to pre-existing myofibres) to support muscle recovery^30,35^. The overall outcome is a significant necrosis that extends beyond the limit of the projectile site itself, mainly due to the damage of skeletal muscle fibre membrane (sarcolemma) that is often associated with large calcium influxes from extracellular milieu, which in turn promotes deleterious effects on intramuscular homoeostasis. Other factors to consider include changes to connective tissue, which have been investigated in detail using laceration models^28,36^. Even relatively small injuries in humans can affect connective tissue and induce fibrosis, leading to scar formation, dysfunction and pain, thus highlighting their importance^31^.

### Acute muscle response to ballistic trauma

One major consequence for muscle viability after ballistic trauma is the severity of mechanic-thermo injury and subsequent myofiber trauma, which is characterised by distinct stages including necrosis, inflammation, and muscle regeneration (Fig. 2) (see ‘*Muscle regeneration*’ section). Mechano-thermo muscle trauma is characterised by abrupt soft-tissue penetration and heat generated by projectile friction, which is accompanied by extensive structural disruption and atrophy of muscle fibres (see ‘*Muscle wasting*’ section), as well as damage to connective tissue, the neural network, and the vascular bed (see ‘*Angiogenesis*’ section). Based on myotoxin/cryolesion models that inflict high levels of sarcolemmal injury, calcium influx likely starts immediately after projectile damage and will extend up to ~30 min, when cellular mechanisms are triggered to initiate the repair process. At this point, changes in tissue morphology in the injured area include disrupted skeletal muscle fibres, where segmented skeletal muscle fibres lose anchoring and shorten to appear enlarged and dense under histological evaluation^37^.

A few studies have employed animal models to directly investigate and characterise thigh muscle reaction and subsequent wound development following firearm bullets or steel projectiles (~1000 m s^−1^)^38^. Following gunshot injury of sheep, it has been reported that hindlimb muscles demonstrated immediate tissue destruction and ischaemia, then at 3 days necrosis developed, which was complete at 6 weeks, while fibrotic scar tissue started at 7 days^39^. In response to projectile missiles, early data (in both swine and canine studies) have also shown skeletal muscle is characterised by severe fibre swelling, a loss of muscle architecture, sarcoplasmic clotting, and severe metabolic derangement (i.e., elevated lactate and depleted high-energy phosphate concentrations)^40–42^. Additional experiments in canine hindlimbs also showed rates of protein synthesis are depressed, indicating a rapid onset of muscle atrophy^40,43^. Further data confirmed that muscle abnormalities are more severe when subjected to haemorrhage^43^, with reduced blood flow and low tissue PO_2_^44,45^, highlighting the importance of vascular function to maintain muscle homoeostasis and protein turnover. Collectively, therefore, ballistic muscle injuries can be characterised by functional loss that is underpinned by necrosis, fibrosis, fatty infiltration, and muscle atrophy. In addition to these, vascular and neural impediments also occur, which highlight a broad range of potential therapeutic targets. Fortunately, many skeletal muscle trauma injuries can be reversed, given adequate time, by endogenous repair processes in the body that require adequate immune, metabolic and neurovascular responses, which involve satellite cell activation to aid muscle fibre regeneration (Fig. 2).

## Skeletal muscle regeneration after trauma

The injury-repair process can be characterised by migration of inflammatory cells to the damaged site, muscle stem cell proliferation and differentiation, followed by maturation of regenerating myofibers, and the restoration of extracellular matrix components (Fig. 2). Altogether, these processes allow recovery of muscle mass and contractile function in many instances^31,46,47^. Skeletal muscle has an outstanding regenerative capacity that is underpinned largely by a rare population of muscle-specific progenitor cells termed satellite cells that have been shown to be required for skeletal muscle repair^48–50^. Satellite cells are located beneath the basal lamina in a mitotically quiescent state and upon injury, such as ballistic trauma, are activated and proliferate into myogenic precursor cells (i.e., adult myoblasts^51^) (Fig. 2). Subsequently these cells either undergo self-renewal to restore the pool of quiescent satellite cells, or initiate a myogenic differentiation programme by fusing with one another and/or with uninjured portions of myofibres to re-establish the damaged areas of the muscle tissue and the myofibre integrity, respectively^52,53^. Unfortunately, at present, most of our understanding of the muscle damage/regeneration process comes from non-ballistic conditions, such as chemical or other physical trauma models (e.g., freeze injury). As such, one must be careful when extrapolating skeletal muscle damage/regeneration from other models in relation to ballistic trauma, in particular from those using chemical/toxin-induced disruption where muscle regeneration will likely follow a very different temporal and spatial response^27,28^. For example, VML is commonplace after a high-energy physical trauma where complete destruction of the local muscle stem cell microenvironment, including the basal membrane, myofiber, and vasculature is accompanied by a localised loss of satellite cells^54^. In contrast, while chemical/toxic injury is associated with less immediate physical destruction of the local muscle stem cell environment, these can have more long-range anatomical consequences that could induce an overall greater loss of satellite cells vs. high-impact physical trauma^54^. Consequently, the regenerative response between physical and chemical/toxic trauma can be very different, with high-energy ballistic trauma promoting a profound loss of local satellite cells and the myofibre milieu that likely requires a greater migration of myogenic progenitors to the site of injury, which may result in an ostensibly delayed regenerative potential than that typically reported.

### Mechanisms controlling satellite cell function

In addition to cytokines secreted by the inflammatory cells, several factors produced by the damaged muscle tissue are able to stimulate satellite cell proliferation, such as fibroblast growth factor(FGF)-6^55^, leukaemia inhibitory factor^56^, hepatocyte growth factor (HGF)^57^, interleukin-6^58^, tumour necrosis factor-α^59^ and insulin-like growth factor I (IGF1)^60^. The myogenic programme is regulated by key transcription factors that command the progression from quiescence, activation, proliferation, and differentiation/self-renewal of satellite cells. Quiescent satellite cells are generally characterised by expression of the paired box 7 transcription factor (Pax7) and Myf5^61^, that upon activation rapidly upregulate Myf5 and start to express MyoD, which lead to the formation of myoblasts along with intense proliferation^52,62,63^. As myoblasts are formed, Pax7 expression declines and myogenin expression marks the onset of terminal differentiation by inducing either auto-fusion or fusion to pre-existing myofibers^52,62,63^. One of the key molecules involved in myocyte fusion is the focal adhesion kinase (FAK), a tyrosine kinase that is activated upon binding of integrins to ligands present in the extracellular matrix. Activated FAK associates with additional proteins that result in the activation of other regulatory pathways such as Wnt, MAPK, Rho guanosine triphosphatases, calcineurin, NF-kB and TGF-β^64^. Ultimately, terminal differentiation is accomplished by activation of sarcomeric and regulatory genes^63^, leading to re-establishment of the structure and function of regenerating myofibers in around 3 weeks after injury^35^. Noteworthy, the Notch signalling pathway has been reported as being essential in maintaining satellite cells in the quiescent state, but is immediately downregulated upon satellite cell activation^65,66^. Interestingly, recent findings now show Notch-regulated quiescence is a highly active and orchestrated process, which involves satellite cell secretion of extracellular matrix collagens (via collagen V-calcitonin receptor signalling^67^), as well as microRNAs (via miRNA-708-Tensin 3 axis^68^). In this regard, it has been shown that quiescent satellite cells can exist in two distinct functional states: deep quiescence (*G*_0_) or primed alert (*G*_Alert_)^69^. Satellite cells in *G*_Alert_ are primed to activate and participate in muscle repair, which can be initiated following exposure to circulating factors such as HGF that have been induced by anatomically distant muscle injuries^70^. This may have important implications for injuries suffered after ballistic trauma given satellite cells in *G*_Alert_ have a greater regenerative capacity^69^. An important mechanism controlling the transition towards this alert state is the kinase mTORC1^69^, with the mTOR pathway being described to be essential for satellite cell homoeostasis and function during muscle regeneration since it controls the expression of myogenic regulatory genes, such as Pax7, Myf5, MyoD and Myogenin^71^. Recently, mTORC2 was also found to be critical for satellite cell maintenance and self-renewal following repeated injuries, supporting the various roles this kinase plays in supporting normal muscle regeneration^72^. Various other molecular regulators of satellite cell function have also been identified, as reviewed extensively elsewhere^51–53,73^. However, despite skeletal muscle regenerative process being well-defined at the structural level, the molecular mechanisms involved are still being intensely investigated and a better understanding of this process is crucial for the development of more effective therapeutic strategies to improve muscle regeneration after firearm wounds.

### Muscle regeneration after mild or severe ballistic trauma

The literature is sparse in regards to the mechanisms of muscle regeneration and satellite cell function following firearm-related injuries. One study showed gunshot injury of sheep hindlimb muscle documented the appearance of regenerating muscle fibres at 7 days to aid fully recovery by 3 months^39^. One major consideration for muscle regeneration following ballistic trauma is the severity of the injury. In contrast to mild injuries (which can rely on endogenous repair mechanism to restore muscle function and mass), a severe injury inflicted by ballistic trauma will include VML and preclude full restoration of muscle mass and function, causing chronic pain and loss of function^74^. Indeed, many firearm wounds are associated with severe VML, which is characterised as an irrecoverable injury associated with severe and permanent loss of muscle mass and function^75^. This injury is associated with limited muscle regeneration, extensive fibrosis and chronic inflammation that accompanies loss of both intracellular contractile proteins and extracellular matrix and longstanding chronic functional deficits^76,77^. VML wounds have major implications as muscle mass cannot be restored by typical muscle regenerative processes, which primarily involve activation of resident satellite cells to support injury-repair and regeneration (Fig. 2). As such, patients afflicted by severe injuries such as VML will, therefore, be reliant on other approaches to aid muscle regeneration rather than usual endogenous processes^74^ (see ‘*Novel strategies to promote muscle regeneration’* section). For example, many low-velocity bullet and mild wounds can heal well by endogenous mechanisms alone without any surgical intervention, resulting in muscle regeneration, discomfort, and functional recovery (mild-moderate injury, Fig. 2), if standard wound treatment procedures are followed (e.g., superficial irrigation, sound cleaning, antibiotic prophylaxis, subsequent outpatient management^9^). However, when a threshold of damage occurs that overwhelms the organism’s intrinsic capacity, chronic pain and loss of muscle mass and impaired function become irrecoverable (e.g., severe injury; Fig. 2). As such, when injuries are of high-energy origin and cause severe tissue damage, aggressive operative procedures are often used that includes early surgical debridement (to remove devitalised tissue) as well as engraftment, where muscle flaps are used to replace large volumes of tissue lost to aid functional recovery. As one of the surgeon’s greatest challenges is treatment of damaged muscle^9^, the development of novel strategies to support skeletal muscle regeneration should be considered a matter of great importance.

## Skeletal muscle atrophy induced by trauma

### Mechanisms controlling muscle mass

**Fig. 3:**
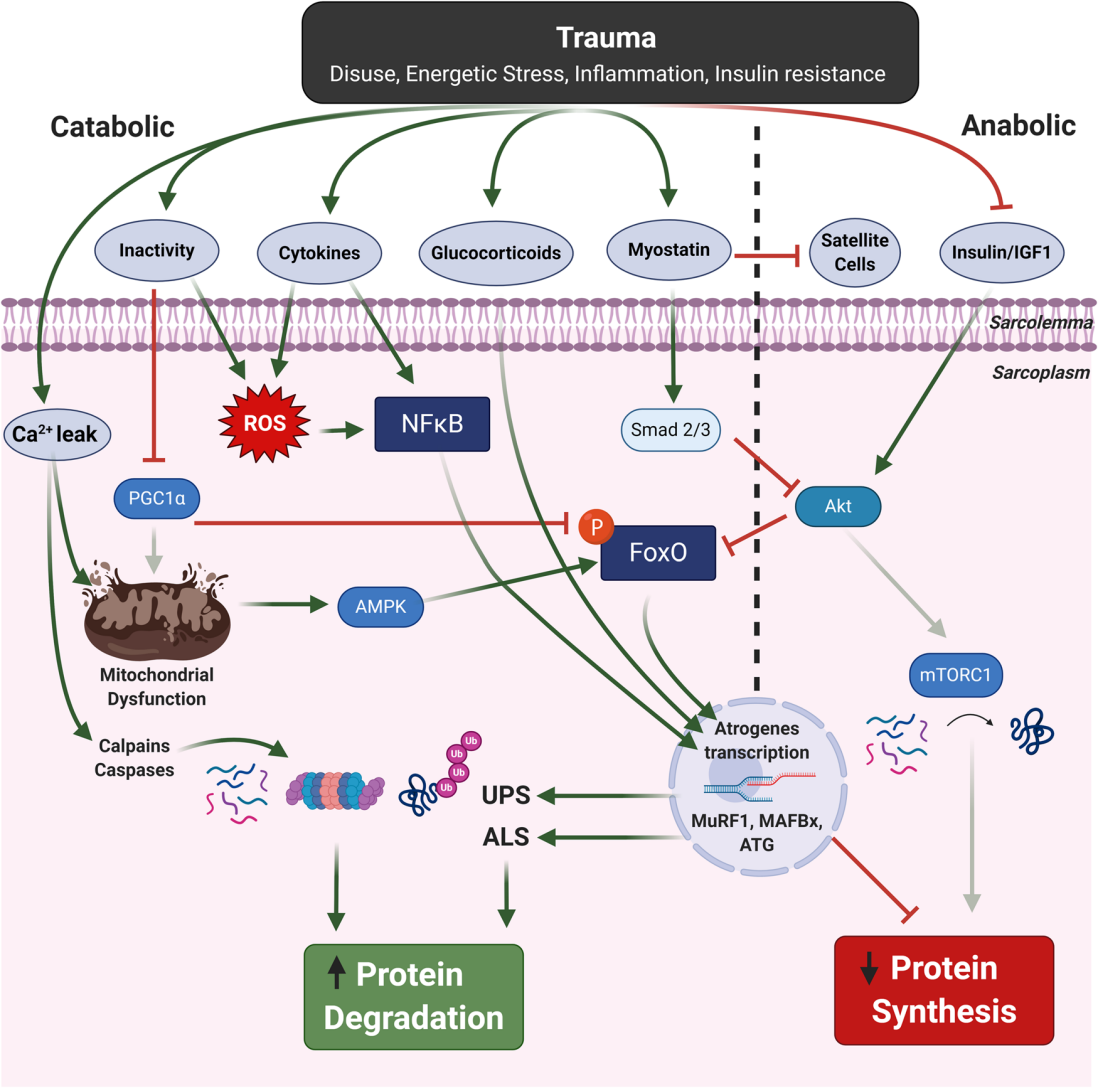
Potential molecular mechanisms mediating muscle atrophy following ballistic trauma. Trauma is associated with various alterations, such as disuse and inflammation, which can induce muscle wasting through various signalling pathways that act to elevate protein degradation (catabolic) and suppress protein synthesis (anabolic). Two key transcription factors regulating muscle atrophy are FoxO and NF-kb, which are activated by numerous upstream factors to promote increases in proteolysis that include the proteasome and autophagy systems. Elevated cytosolic calcium (Ca^2+^) levels can also increase calpain and caspase proteolytic activation alongside impairing mitochondrial function. See main text for expanded details.

As discussed earlier, an important consequence of ballistic trauma is the loss of muscle mass, which is a major factor determining functional status, quality of life and mortality^78^. Muscle mass is determined by the complex balance between rates of protein synthesis and degradation (Fig. 3), where a shift in either can result in loss or gain of mass^79^. An imbalance between protein anabolism and catabolism is often modulated by upstream factors related to changes in hormones/growth factors, mechanical loads, neural activation, and cellular energetic status. As such, changes related to activity (i.e., disuse), inflammation, hyperglycaemia/insulin resistance, intracellular calcium concentrations, or energetic stress (i.e., reduced ATP/AMP ratio) are major signals that can initiate muscle wasting following ballistic trauma (Fig. 3). On one hand muscle wasting can result from reduced protein synthesis due, in large part, to downregulation in one major signalling pathway related to the insulin/IGF1-Akt-mTORC1 pathway as this regulates protein translation^80^. However, wasting can also be caused by an increased rates of protein degradation, which is mediated by two principle systems termed the ubiquitin proteasome and autophagy-lysosome that work alongside two calcium-dependent pathways consisting of the calpain and caspase proteases that are able to cleave target proteins^80,81^. These atrophic systems are largely controlled by a subset of highly regulated transcriptional factors that can induce proteolytic activity, with the forkhead box protein O (FoxO) and NF-kB transcription factors central^80,81^. These catabolic transcriptional regulators control the gene expression of key muscle-specific E3 ligases, which repeatedly label targeted proteins with ubiquitin. Polyubiquitinated proteins are thereafter recognised and degraded via the 26S proteasome complex as part of the ubiquitin proteasome system. In general, the ubiquitin proteasome system is considered the major proteolytic pathway in the fibre responsible for degrading sarcomeric contractile proteins^82^. Therefore, E3 ligases are thought to represent a rate-limiting step in the wasting process. Two important E3 ligases shown to be upregulated across a wide range of wasting conditions, and key for atrophic induction, are MAFbx and MuRF1^81^.

The other major proteolytic system involved in wasting, which is also regulated by FoxO transcription, is the autophagy-lysosomal pathway. This pathway targets damaged organelles for removal, such as the mitochondria (i.e., mitophagy), by forming autophagosomes that subsequently undergo lysosomal degradation^80,83^. Indeed, mitochondrial number, shape and activity is extensively remodelled in muscles undergoing atrophy, with stimulation of mitochondrial fission shown to induce muscle atrophy and conversely inhibition preventing muscle mass loss^83^. While the mitochondria were initially assumed to be simply energetic organelles, recent evidence show they play a key role in regulating important signalling molecules, which in turn, control muscle wasting. These include reactive oxygen species (ROS), the energetic sensor AMP protein kinase (AMPK), and the master regulator of mitochondrial biogenesis nuclear transcriptional co-activator peroxisome proliferator-activated receptor-gamma co-activator-1α (PGC-1α)^83^ (Fig. 3). Other novel factors recently linked to atrophy that are related to mitochondrial function and energy homoeostasis include the fission/fusion proteins Mitofusin 2 (Mtf2)^84^, Drp1^85^, and OPA1^86^, while the family of stress-inducible metabolic regulators sestrins have also recently been identified to have dual effects on both anabolic (i.e., Akt-mTORC1) and catabolic (i.e., autophagy-proteasome) control mechanisms^87^. Other important hormones/growth factors secreted under catabolic conditions include myostatin and glucocorticoids. Myostatin is a member of the transforming growth factor beta (TGF-β) superfamily and a negative regulator of muscle mass, which acts via phosphorylated Smad2/3 to inhibit Akt signalling, as well as inhibiting satellite cell function^88^. Glucocorticoids on the other hand are steroid hormones, and despite being commonly used to treat inflammatory conditions, they are potent mediators of muscle wasting via activation of the glucocorticoid receptor transcription factor, which acts to increase FoxO and MuRF1 transcription in parallel to inhibiting mTORC1 activity to suppress protein synthesis^80^. Importantly, there is a close crosstalk between anabolic and catabolic signalling pathways: for example the protein kinase Akt can suppress atrophy via inhibiting FoxO signalling^89^ (Fig. 3), although of interest a more complex interplay between the pathways has recently come to light such that sustained mTORC1 activation has been linked to muscle wasting during disuse atrophy via disruption of homoeostatic autophagy^87^. Overall, our molecular understanding of many conditions characterised by muscle wasting (e.g., immobilisation, cancer, heart failure, COPD, sepsis) has extensively improved over the last few decades, showing mechanisms related to protein synthesis are suppressed alongside rates of protein degradation being elevated^80^.

### Potential mechanisms of muscle wasting after ballistic trauma

Immobilisation, systemic inflammation, hyperglycaemia/insulin resistance, and nutrient deficiency are common side effects induced by ballistic trauma^7^, which are well-established mediators of muscle wasting in both the acute and chronic setting^90^. However, in regards to ballistic trauma alone, the molecular mechanisms modulating muscle mass remain poorly explored. Thus, most of our knowledge so far is derived from alternative models that include chemical-thermal injury (i.e., freeze injury, barium chloride, notexin and cardiotoxin), physical damage (i.e., VML, contusion, laceration, ischaemia, eccentric physical exercise) and chronic models mainly related to myopathies^26^. One major hurdle in humans in relation to ballistic trauma is the limitations in experimental design, while there remains a scarcity of research in translational animal models following firearm-related muscle injuries. Early studies utilising swine and canine traumatic models revealed gunshot projectile damage to thigh muscle resulted in a suppression of protein synthesis (as measured by leucine incorporation), showing both regional and time effects^40,43^: muscle protein synthesis was suppressed to the greatest extent soon after injury and in the region closest to the site of injury. Additional experiments in the pig hindlimb showed that ribosomal activity (an index of protein synthesis) was also depressed, and this was the greatest in combination with haemorrhage^43^. These data reinforce the important role vascular function plays in regulating muscle mass following trauma, while it has been shown that projectile damage induces very low levels of PO_2_^45^ and blood flow^44^ in skeletal muscle. Collectively, while these data support protein synthesis depression following firearm trauma, little data have been collected in regards to changes in proteolysis or the precise molecular regulators involved. Below, as shown in Fig. 3, we propose a number of key mechanisms that likely induce muscle wasting after firearm-injury which include:

1. *Energetic and calcium imbalance:* The energy state of the cell (i.e., low ATP/AMP ratio) is well known to exert control over muscle mass, via activation of the protein kinase energy sensor AMPK, which increases FoxO signalling and thus subsequent proteasome and autophagosome-dependent protein degradation^91^ (Fig. 3). Conversely, AMPK has also been shown to block mTORC1 activation and can thus suppress protein synthesis as well^79^. Given the clear metabolic perturbations reported to occur in skeletal muscle following ballistic trauma (e.g., shift to anaerobic metabolism and lower concentrations of high-energy phosphates^40–42^), AMPK would likely be activated in this setting. Another key mechanism that would likely promote muscle atrophy following ballistic trauma is activation of the key proteolytic calpain and caspase systems, which are increased by elevated cytosolic calcium concentrations (Fig. 3). A significant increase in cytosolic calcium flux is common following muscle trauma and this event, which can also induce mitochondrial dysfunction and ROS production^83^, is likely a key trigger for elevating proteolytic calpain and caspase activities to initiate cleavage of sarcomeric proteins for subsequent degradation (e.g., caspase-3 targets the actomyosin complex)^92^. In fact, data now show that attenuating disturbances in cytosolic calcium (i.e., leak) under stress conditions, as observed in conditions such as critical illness, is associated with preserved muscle mass and function^93^.
2. *Inflammation and hormonal disturbances*: one major consequence of projectile trauma is an inflammatory response, which is increased following high-energy penetrating wounds to the extremities and leads to a severe elevation in systemic cytokines (i.e., IL-6, IL10, MCP-1)^94^. Often infection and subsequent development of sepsis is commonly developed in trauma, which further exacerbates inflammation and muscle wasting^95^. Although there seems to be a lack of data on firearm projectiles on skeletal muscle atrophy related to the pro-inflammatory state, many in vitro studies alongside in vivo patient studies during critical illness have shown that TNF-α, IL-6, or IL1β can directly activate the ubiquitin proteasome pathway via the transcription factor NF-kb in a MuRF1-dependent manner^95^ (Fig. 3). While inflammatory cytokines may reduce muscle mass, they can also induce contractile dysfunction (i.e., impair force generation normalised for muscle mass by disrupting excitation-contracting coupling), which would further exacerbate muscle weakness following ballistic trauma. In addition glucocorticoids concentrations are elevated under stress and could induce wasting following ballistic trauma in a MuRF1-dependent manner in parallel to supressing protein synthesis via downregulation of mTORC1^79^. Furthermore, the secreted growth and differentiation factor myostatin is known to be elevated following trauma^96^, which would be predicted to induce atrophy and blunt regeneration after ballistic trauma by means of: (1) blocking Akt activity to reduce protein synthesis while elevating FoxO-dependent atrogene transcription; and 2) blunting satellite cell proliferation and differentiation to impede regeneration^97^.
3. *Insulin resistance and disuse:* hyperglycaemia and insulin resistance can develop rapidly after trauma, which can impair IGF1/insulin-Akt signalling to suppress protein synthesis whilst in parallel activating FoxO transcription of key atrogenes that elevate proteolysis^95^. Immobilisation is often a consequence of many traumatic injuries, which leads to severe muscle wasting. Interestingly, this is suggested to occur via suppression of protein synthesis alone (rather than impacting proteolysis) and be independent of Akt signalling^98^. Evidence indicate that a reduction in muscle contractions per se also drive the development of subsequent insulin resistance, and in combination with a systemic inflammation exacerbates insulin insensitivity to enhance muscle wasting by both a reduction in protein synthesis and elevation in degradation^98^. In addition, critical care patients often receive drugs such as corticosteroids and neuromuscular blocking agents that also have catabolic wasting effects^95^.
4. *Reactive oxygen species:* one unifying mechanism for how inflammation, hyperglycaemia/insulin resistance, and immobilisation elevate muscle atrophy is via increased levels of ROS^99^ (Fig. 3). Elevated ROS can directly activate proteolysis (e.g., via redox-sensitive NF-kB transcription, autophagy, calpain, caspase-3) and inhibit protein synthesis (e.g., via Akt/mTORC1 blockade)^99^, while also damaging intracellular proteins related to myofibrils, sarcoplasmic reticulum-calcium handling, and energy metabolism to induce contractile weakness and fatigue^99^. One major source of ROS is the mitochondria, which is a central mechanism driving muscle atrophy^99^ and this seems to be mediated in part via a calcium leak-calpain mediated mechanism^100^. Acute hypoxia is also known to be a potent stimulus of ROS and subsequent muscle dysfunction^101^, thus further highlighting the key role normal vascular function plays in muscle homoeostasis, which we will now discuss.

### Role of angiogenesis in skeletal muscle trauma

**Fig. 4:**
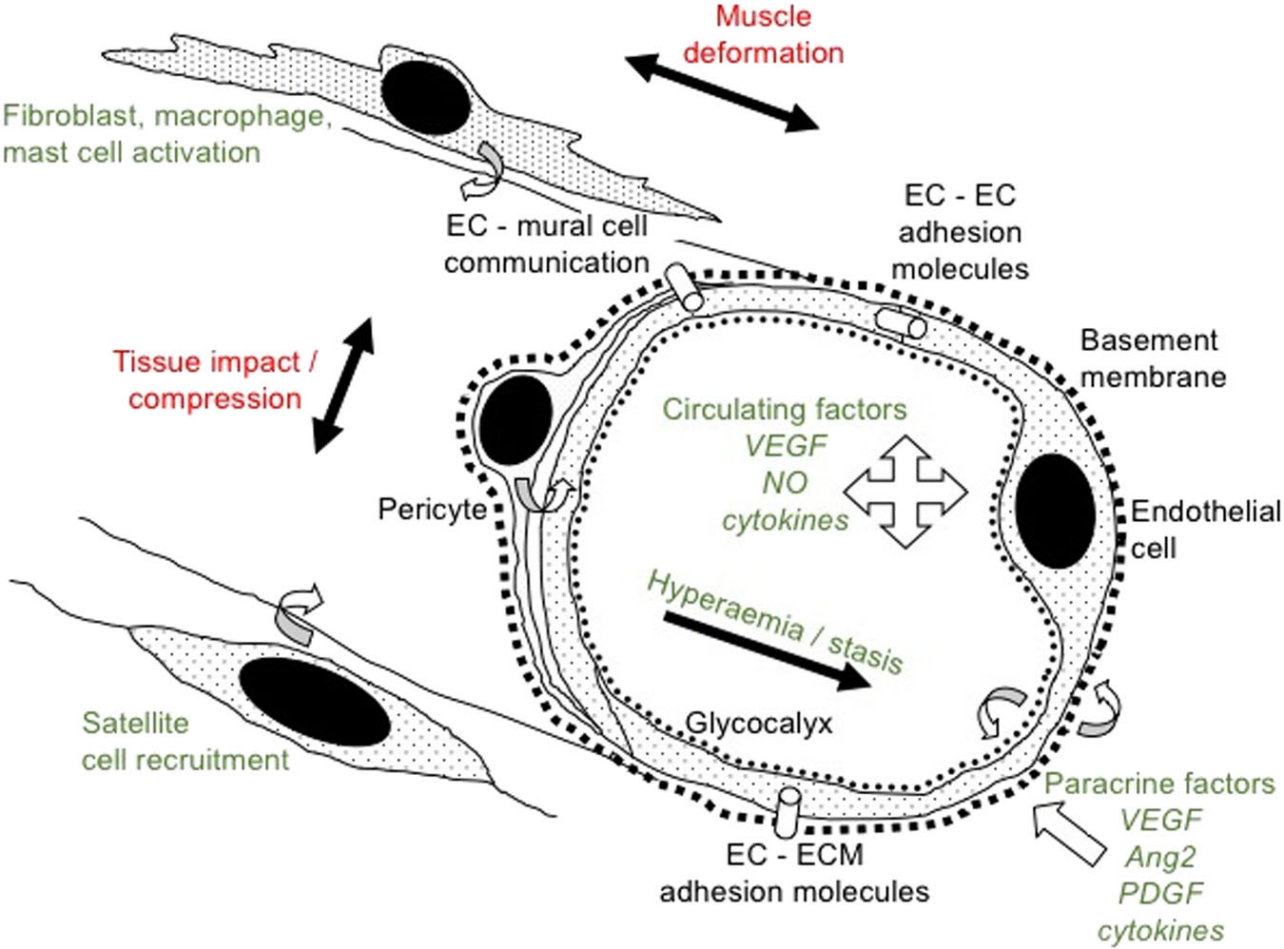
Overview of mechanisms involved in regulating vascular function and angiogenesis. Expansion of the microcirculation may be elicited by a number of discrete or complex stimuli, involving independent or synergistic mechanisms. These include mechanical factors (either sensed directly by cells or indirectly via the extracellular matrix, ECM) involving initial tissue compression and haemostasis, and subsequent muscle deformation and hyperaemia (e.g., wall strain, shear stress). Activation of resident or infiltrating mural cells (e.g., macrophages, mast cells), either directly due to mechanical trauma or indirectly by the following cytokine storm, may in turn activate endothelial cells (EC). The efficacy of other cell types important for muscle regeneration, such as satellite (mesenchymal stem) cells, appears to be related to their proximity to capillaries; perivascular pericyte communication with EC may modulate the response. Finally, chemical signals from remote or local paracrine sources (e.g., cellular release or cleavage from ECM-binding sites) complete the repertoire required to free EC from their usual quiescence. Figure is adapted from Egginton^106^.

Following ballistic trauma one major factor is haemorrhage, however, with advances in medicine this is now often controlled by rapid volumetric replacement that stabilises blood homoeostasis and pressure^90^. However, angiogenesis is critical not only for wound healing but also skeletal muscle homoeostasis, important for preventing muscle atrophy and supporting satellite cell function (Fig. 4). The term angiogenesis refers to growth of the smallest blood vessels, capillaries/exchange vessels, from pre-existing vessels (cf vasculogenesis, the de novo generation of new vessels, such as embryogenic neovascularisation); angioadaptation describes capillary growth and regression, involving integrated responses among vascular cells and host tissue, reflecting an interplay between mechanical and chemical stimuli^102^. The importance of capillaries cannot be understated, being essential for substrate/O_2_ delivery and metabolite removal from active tissue, as well being an essential component of the endocrine system. There are at least 20 angiogenic growth factors that aid stimulation of new blood vessel growth, and others that prevent excessive growth, which help restore blood flow and enhance oxygen diffusive exchange to ischaemic heart, limbs, and brain; and to heal wounds^103–105^.

Studies suggest that many firearm wounds of the extremities are associated with vascular trauma^9^, highlighting the prevalence of direct damage. Important control mechanisms lie in endothelial cell (EC) biology and changes with pathology (Fig. 4). Penetrating trauma will impair blood supply, both from physiological vasoconstriction (a powerful sympathetic drive to prevent exsanguination) and physical disruption of the vascular network. Widespread ischaemia will lead to tissue hypoxia, with subsequent acute allosteric and chronic transcriptional responses orchestrated by key regulatory hub genes such as HIF-1α and PGC-1α inducing, among other cytokines, peptide growth factors such as vascular endothelial growth factor (VEGF). Interestingly, it may not be the absolute level that is important, but the link with expression of cognate receptor(s)^106^. Providing anoxia is avoided, the microcirculation appears to be quite resilient (likely due to the high glycolytic capacity of EC), and capable of regrowth. Delayed recovery of O_2_/substrate delivery will lead to regression by apoptosis, and rarefaction of the capillary bed in a coordinated (non-random) manner. Tissue function has been shown to correlate well with functional capillary density, emphasising that angiogenesis is an appropriate therapeutic target. However, for a short period after capillary regression remnants of the basement membrane sleeve appear to offer a preferential channel that aids vessel regrowth, explaining the need for rapid onset of rehabilitation^107^. During recovery, the survival of any muscle graft depends on acquiring an adequate blood supply before it dies – the window of opportunity being inversely proportional to graft metabolic requirements, its bulk and original vascularity.

## Novel strategies to promote muscle regeneration after ballistic trauma

At present the most common method for regaining muscle mass and function following ballistic or blast injuries is, as with most trauma injuries, via physical rehabilitation. However, we currently lack effective therapies that can aid optimal muscle regeneration and recovery immediately after projectile trauma. Preclinical studies have shown that even following severe muscle loss (i.e., VML), treadmill running results in functional benefits^108^, although optimised frequencies, durations, intensities remain poorly explored. Interestingly, delaying the onset of an exercise regime may provide more benefits than initiating exercise too early post-trauma^109,110^ although evidence varies^111^. Indeed, following projectile impact, it is important to establish a period of inactivity to prevent further tissue impairment by contraction of the damaged area, and aid pain control. However, as discussed earlier, limb immobilisation can result in disuse atrophy via suppression of protein synthesis^98^. Thus, the balance between adequate rest and re-starting muscle activity must be taken into account, and further research is required to optimise clinical management. Another major approach for enhancing muscle recovery after ballistic trauma includes surgical intervention, such as muscle grafts, while standard approaches in the field include conservative management (RICE principle; rest, ice compression and elevation), standard medication (e.g., non-steroidal anti-inflammatory drugs; NSAIDs), and direct muscle stimulation^21^. Other approaches have also included light and acoustic waves, as well as topical negative pressure following wound injuries^7^. While these approaches may help restore some muscle mass and function, reduce fibrosis, and activate angiogenesis following moderate trauma, their outcome remains highly variable and often unsuccessful in patients with the most traumatic wounds such as VML^74^.

**Fig. 5:**
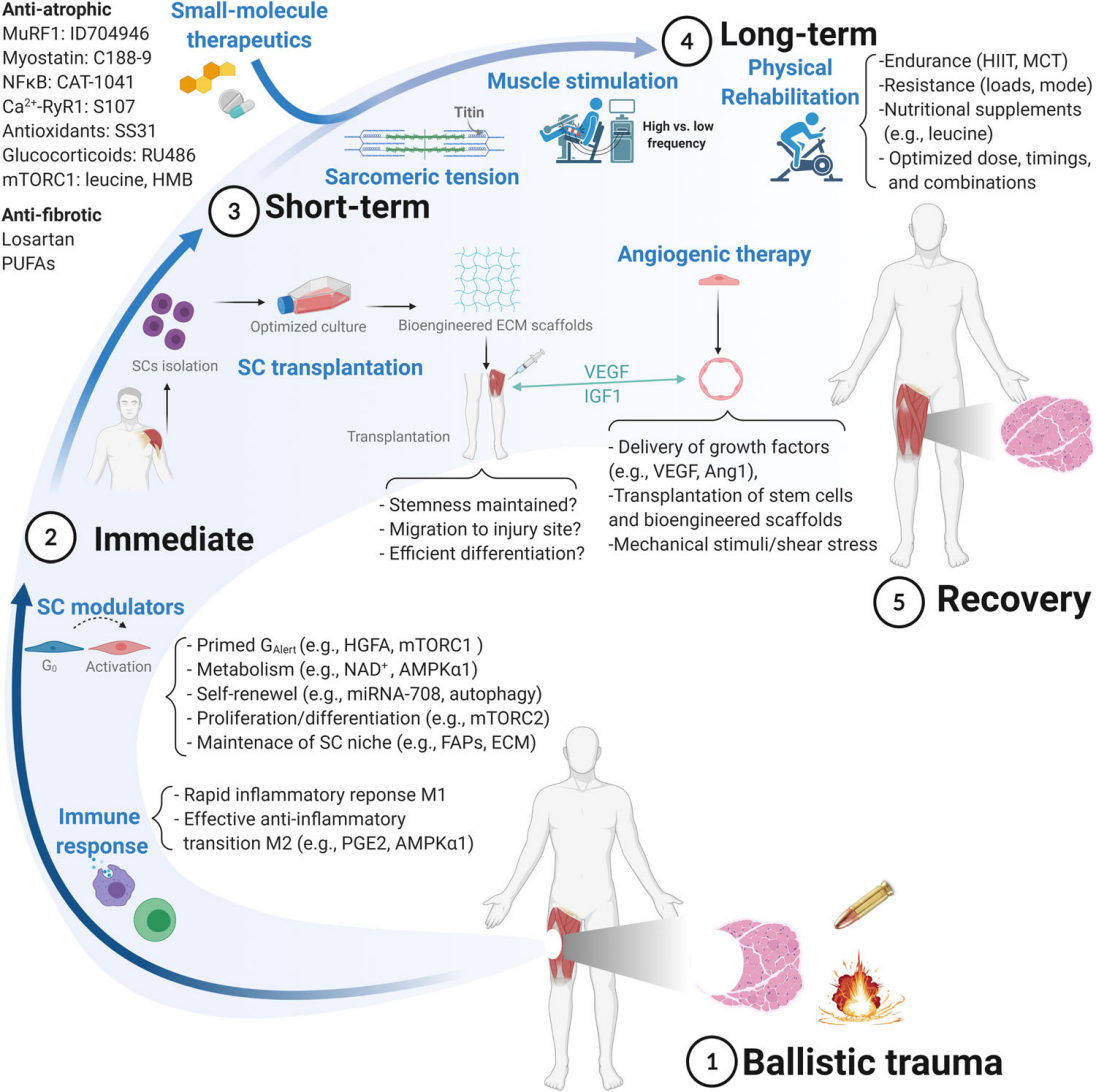
Putative mechanistic targets and strategies for improving skeletal muscle regeneration after ballistic trauma, where volumetric muscle loss (VML) is a common consequence. After ballistic insult (1) various time points can be targeted to enhance muscle regeneration, which include immediately post injury (hours-days) (2), the short-term (days-weeks) (3), and the long-term (months-years) (4), with the potential outcome being full or at least partial recovery (5). Emerging interventions/targets exploiting the intrinsic properties of both immune and satellite cells (SCs) during the initial response phase to injury could accelerate the repair process (1). Beyond the immediate endogenous response, short-term strategies could involve muscle and angiogenic stem cell therapies, while the administration of myofibre-targeted novel small-molecule therapeutics could block catabolic/fibrotic activity while promoting anabolic signalling, whereas other emerging therapies include focussing on sarcomeric mechano-dependent signalling to maintain muscle mass (2). Technological advances have allowed more effective in vitro culturing of isolated SCs by closely recapitulating the stem cell niche and, when combined with biomaterials for transfer, allow for a more efficient transplantation and muscle regeneration capacity although a number of technical challenges still remain (3). Angiogenic therapies can be used to complement muscle regeneration, which stimulate vascular growth whereby a close cross-talk via local factors between endothelial cells and SCs can enhance muscle regeneration. Long-term, electrical muscle stimulation could be incorporated with varying frequencies to mediate myofibre plasticity and reinnervation, while physical therapy forms the mainstay of any extended rehabilitations (4). Improvements associated with exercise training will largely be dependent upon the specific regimes employed, such as high-intensity interval training (HIIT) or moderate-continuous training (MCT), the low- or high-resistance training exercise loads, as well as selecting the optimal combination of exercise and nutritional supplements for maximal physiological benefits. Overall, a combined holistic approach such as cell therapy, anti-catabolic small molecules, and physical exercise would likely provide the most effective approach for improving muscle regeneration and aiding rapid functional recovery (5). See main text for full details of putative interventions and supporting references.

At present, we currently lack effective therapies that can aid optimal muscle regeneration and recovery immediately after projectile trauma. While current deployed trauma care has reached a high level for both civilian and military human populations, there is still a major limitation in reversing damage^7^. Thus, more innovative approaches are urgently required. One area that holds promise is that of regenerative medicine, with key advances for treatment reported in terms of satellite cell therapy, small-molecule therapeutics for anti-catabolic and pro-anabolic agents, and delivery of angiogenic growth factors (Fig. 5). In particular, it has been recently highlighted that the ability to start regenerative treatment at a very early time point following ballistic trauma (i.e., on the geographical location) would likely provide the greatest step change in current trauma care, with the risk of permanent injury during ballistic wounds suffered in military conflict highly associated with post hoc care^7^.

### Satellite cell therapy

Given skeletal muscle regeneration after injury is highly dependent upon resident adult muscle stem cells, these form a major putative therapeutic target after ballistic muscle wounds. Indeed, satellite cells alongside muscle resident cells with multi-lineage potential (such as muscle-derived stem cells, pericytes and mesoangioblasts) and cells with non-myogenic origin (such as mesenchymal stromal cells from bone marrow or adipose tissue and induced pluripotent stem cells) contribute to muscle repair, either by participating in new tissue formation or by inducing endogenous muscle repair^26,112^. However, as many ballistic wounds are associated with severe VML, muscle mass cannot be restored by endogenous muscle regenerative processes due to loss of the satellite cell pool. Traditional approaches for VML have, therefore, typically included the placement of muscle flaps and removal of scar tissue, although this has been met with limited success^113^. As such, other advances have included autologous minced muscle grafts (which contain essential components for muscle regeneration)^114^, with one of the most promising approaches being the transplantation of isolated satellite cells^115^. Despite enthusiasm, however, various limitations during the transplantation process have been encountered that have conflated to yield compromised engraftment capacity, which have included problems related to in vitro expansion, maintaining intrinsic stemness properties, survival post-transplantation, and limited differentiation ability and migration towards the site of injury^116^. Therefore, further work needs to be done to optimise current stem cell strategies for more efficient muscle regeneration.

Notwithstanding the aforementioned, various innovative approaches have been proposed that include altering chemical and physical properties of biomaterials to improve cellular function^74^, thus improving biomaterial design to deliver and retain viable cells near the damaged region and engineering a sufficient niche^26,109,117,118^. Indeed, research has therefore been focused on developing tissue engineered therapeutics that could be used to restore muscle quantity, including the use of scaffold and bioengineered constructs^109,119^ and autologous minced muscle^114^. These advances in technology have allowed engineered bioconstructs to be developed^74^, which comprise of muscle stem cells and muscle resident cells (isolated from healthy tissue) alongside extracellular matrix scaffold (decellularised muscle), and implanted within injured muscle (Fig. 5). These studies have shown benefits in animal models subjected to VML (following removal of ~40% of the *tibialis anterior* muscle, at which point scaffold and bioconstructs were implanted, and muscle closed). In particular, the bioconstructs resulted in de novo myofiber formation in vivo, near normalisation of muscle mass, and rescued strength alongside reducing fibrosis at only one-month post injury^109,119^. It is important to highlight that bioconstructs implanted with stem cells alone had limited benefit and only when scaffolds were transplanted with both stem cells and muscle resident cell population were the greatest effects seen^109^, suggesting targeting both intra- and extracellular components is required. In this regard, therapeutic approaches that fully utilise the myofibre niche should be seen as essential to fine-tune and accelerate muscle regeneration^52,53^, especially for VML where large parts of the muscle milieu are lost after a ballistic wound. For example, in vivo intravital microscopy experiments have confirmed that ‘ghost fibre’ remnants of damaged myofibers act as scaffolds to direct satellite cells towards the site of injury and support muscle regeneration following cardiotoxin-induced injury^120^, while another study showed that engineered ECM scaffolds with parallel microchannels enhanced endogenous muscle and vascular repair^121^. Indeed, endothelial cells are necessary in the process to sustain satellite cell function, again suggesting maintenance of vascular homoeostasis is critical for the muscle regeneration process (Fig. 4). Thus, while simple transplantation of muscle progenitor cells alone is likely beneficial, incorporation of the satellite cell and myofibre niche seem requisite for maximum benefit. Importantly, it has been shown that the addition of physical exercise in combination with constructs provides the greatest benefits, due to effects on innervation (which constructs do not achieve)^109^ and potentially on restoring Cyclin D1 to aid muscle stem cell activation^122^. While implanted human stem cells have shown similar effects in mice to restore muscle mass^109^, more work still needs to be done to confirm transplantation efficiency in humans given most studies have been performed on mice, while other problems related to cell expansion, autologous harvesting, optimal dosage, and viability after transplantation still need addressing^26,112,123^. Although on a more encouraging note, a couple of studies using a small number of humans with VML (*n* = 5–13) have confirmed partial rescue of muscle mass and function following transplantation of acellular scaffolds derived from porcine urinary bladder^124,125^, which reinforces the potential for this biotechnology as a treatment for humans post ballistic trauma.

Looking at other traumatic injuries (e.g., laceration or tibial fracture model) and towards druggable targets, the anti-hypertensive drug losartan, an angiotensin receptor inhibitor, was able to enhance the number of regenerating fibres and reduce fibrosis and fibre atrophy, effects enhanced in combination with late (not immediate)-initiated physical training^110,126^. However, increased muscle regeneration or mass was not improved in the non-recoverable VML experimental model post-losartan treatment^127^, which highlights that unless combined with scaffold and bioengineered constructs then the efficacy of this drug following firearm trauma would likely be limited. Interestingly, as the quiescent state in satellite cells represents a highly regulated process, the opportunity to exploit this by manipulating satellite cells towards the “primed” state (i.e., from *G*_0_ to *G*_Alert_)^70^ for more immediate activation could be utilised to aid repair^70^. For example, this could be done by utilising novel drugs to prime satellite cells that target various mechanisms, either just before potential injury (i.e., during military battles) or immediately after, with evidence identifying HGFA^70^, mTORC1^69^, NAD^+^-SIRT1^128^ AMPKα1^128^, and/or FoxO signalling^129^ as possible targets. Alternative approaches could include targeting an appropriate and rapid inflammatory response to injury, as the acute treatment with the metabolite prostaglandin E2 (PGE2) has been shown to improve muscle regeneration^130^, while improving the transition towards an anti-inflammatory state during the injury response process has also been shown to aid muscle regeneration via modulation of key targets related to macrophage repair that have included AMPK^131^, the myokine meteorin-like (Metrnl)^132^, and/or p38 MAPK inhibitors^133^. Beyond this and as mentioned above the supporting role of the stem cell niche has become a critical consideration to aid normal muscle repair, via the direct interaction with (or release of soluble factors from) other neighbouring stem cell populations and the interacting extracellular matrix proteins^73^. For example, neighbouring fibro/adipogenic progenitors (FAPs) can release important factors that support and enhance myogenesis during specific stages of the repair process^134^, while mimicking real-world tissue stiffness (i.e., 12kPa)^135^ and encapsulating the extended 3D niche environment within implanted acellular scaffolds promotes more efficient migration, maintenance and differentiation of stem cells^136^. In addition, the development of new techniques such as intravital 3D bioprinting has established the possibility for complex reconstruction inside tissue of living organisms that has been shown to further aid de novo fibre formation^137^, while reprogramming of other cell types (e.g., fibroblasts) towards expandable induced muscle progenitors cells may be another option to enhance the transplantation and regenerative process^138^. Overall, multiple and exciting treatments for muscle dysfunction after ballistic trauma are on the horizon, where in vivo treatment of injured muscle with scaffolds and cells in combination with exercise will likely be optimal (Fig. 5).

### Inhibition of muscle atrophy via small-molecule therapeutics

At present there are no established drugs to rescue muscle wasting in humans^78^, with exercise training the only proven therapeutic treatment^139^. Given ballistic wounds cause immobilisation, metabolic dysregulation, and a systemic inflammation that collectively trigger muscle wasting, the development of novel in vivo drugs to hamper muscle atrophy immediately after trauma and during rehabilitation in immobile patients would represent a major breakthrough. This could be achieved by either attenuating catabolic signalling and/or activating anabolic signalling molecules^81^ (Fig. 5). In support, studies have identified a novel in vivo small-molecule compound capable of attenuating muscle wasting and force loss in experimental cardiac failure (induced by surgical or toxic injury) via inhibition of the key atrophic E3 ligase MuRF1^140,141^, and more recently in cancer cachexia^142^. High-throughput technology screened over 100,000 compounds for their ability to inhibit MuRF1’s interaction with the sarcomeric protein titin, by targeting MuRF1’s central coiled-coil domain^141^. A compound termed ID#704946 was shown to inhibit MuRF1 E3 ligase activity in vitro and when translated to an in vivo wasting model following cardiac trauma, the compound inhibited skeletal muscle MuRF1 expression, proteolysis, contractile dysfunction, and fibre atrophy in skeletal muscle^141^. Exploratory proteomics and further experiments demonstrated the novel compound normalised pathways related to protein synthesis, apoptosis, and mitochondrial function/content^140,141^. Thus, application of novel in vivo MuRF1 inhibitors could offer a viable treatment of atrophy induced by ballistic trauma injuries and further exploration is warranted (Fig. 5). Other small-molecules worthy of further investigation (which may aid muscle growth alongside supporting satellite cell function) include those that inhibit myostatin, such as small-molecule inhibitor C188-9^143^ or anti-myostatin antibody (ATA 842)^144^, with both treatments rescuing muscle wasting induced by chronic kidney disease and ageing. In addition, a novel class of orally bioavailable NFκB inhibitors (termed edasalonexent and CAT-1041) were shown to inhibit muscle wasting in both murine and canine models of muscular dystrophy^145^, which could also be viable for trauma injuries. Another class of small molecules termed microRNAs have also been considered as potential therapeutic devices in skeletal muscle plasticity. Particularly related to muscle mass control, microRNAs 29c and 208 are involved in hypertrophy^146,147^. On the other hand, microRNAs 23, 27 and 486 have been implicated in attenuation of skeletal muscle mass loss^148–150^. Beyond this, data have clearly shown limiting disturbances in cytosolic calcium homoeostasis (i.e., by preventing leak via the Ryanodine Receptor 1; RyR1) during critical illness can prevent muscle weakness and wasting via the drug S107 via attenuating the depletion of the subunit calstabin^93^.

Interestingly, rather than activation (like the proteasome system) the other major proteolytic pathway of autophagy is reported to be suppressed in critical illness and sepsis^95^. While over-activation of autophagy can induce wasting, suppression can also cause similar effects due to its role in maintaining protein quality control^79^. Hence, medical drugs activating autophagy may be helpful in maintaining muscle mass after ballistic trauma by alleviating accumulation of damaged organelles or proteins such as the mitochondria (e.g., as recently shown for sestrins^87^). Given mitochondria’s key role in modulating muscle mass^83^, preventing dysfunction of this organelle could represent a major therapeutic target for rescuing muscle loss in ballistic trauma patients. In support, recent data have shown the PGC-1α signalling axis is disrupted in mice with VML, and that forced expression of PGC-1α using transfection partially rescued muscle strength and oxidative muscle function^151^, which reinforces previous data during disuse atrophy^152^. These data highlight, for the first time, how targeting the mitochondria may play a key role in muscle remodelling post VML. We also know increased mitochondrial ROS production is a key trigger for the induction of muscle wasting in various conditions^99^. Emerging evidence now shows how mitochondrial-specific antioxidants (e.g., mitoQ and SS31) can dramatically reduce muscle impairments in various conditions such as denervation, immobilisation, and hyperglycaemia^99^. For example, a recent study showed how treatment with SS31 (a novel cell-permeable antioxidant that locates to the mitochondria in high concentration) could prevent acute muscle weakness induced by sepsis in mice, which was associated with inhibition of proteasome and calpain activation^153^ and further linked to a calcium RyR1 leak-calpain mediated mechanism^100^. In addition, other mitochondrial-related factors recently linked to atrophy may also represent viable options for future manipulation, which include Mitofusin 2 (Mtf2)^84^, Drp1^85^, OPA1^86^, and/or sestrins via their effects on PGC-1α signalling and autophagy/mitophagy maintenance^87^. However, in relation to firearm-related muscle injuries, little is still known regarding the effects on mitochondrial function and ROS production but further studies are clearly warranted. More experiments are also required to confirm the benefits of mitochondrial antioxidants in humans following trauma or other wasting conditions, given some studies have shown benefits in vascular tissue^154^.

It has recently been highlighted that the biochemical environment during wound healing following ballistic trauma is likely key for success^7^. As discussed, ROS are often increased by the elevated circulating pro-inflammatory cytokines. Thus, some studies have also used novel anti-inflammatory drugs to improve recovery. Key interventions to reduce systemic inflammation in various wasting disorders such as cancer, via targeting TNF-α using Etanercept/Infliximab, for example, have so far been largely unsuccessful, or even detrimental in humans trails^78^. Thus, further work or alternative inhibitors are required to clarify the benefit of suppressing these upstream targets for trauma injuries. Other work, albeit preclinical, has shown more promise when using mild anti-inflammatory treatments, where orally administered fish oil-derived omega-3 poly-unsaturated fatty acids (PUFAs) in combination with high-protein supplements prevented muscle atrophy alongside attenuating fibrosis in chemically induced muscle wasting, suggesting this may be useful for ballistic trauma^155^. Of interest, recent data have also confirmed that FAPs residing in the muscle stem cell niche are not only crucial to support regeneration but their absence leads to the onset of muscle atrophy^156^, supporting FAP maintenance is key. Although seemingly they play a double-edged role, as persistent accumulation of FAPs leads to fibre atrophy and enhanced fibrosis during denervation via an IL-6-Stat3 signalling axis^157^.

Another approach to rescuing muscle loss in ballistic trauma could also be activating anabolic signalling molecules (Fig. 5), which will not only stimulate muscle protein synthesis but via crosstalk will inhibit pro-catabolic factors (e.g., FoxO). Studies have shown conditional activation of Akt in transgenic mice induces muscle hypertrophy without activation of satellite cells^158^, which may be relevant for ballistic trauma in the event of mild injury. However, we currently lack targeted in vivo drugs to activate Akt. Another key node in protein synthesis is mTORC1, which can be activated by growth factors (i.e., IGF1, insulin), nutrients (i.e., leucine), and mechanical load^159^. Data have shown oral doses of the amino acid leucine or its metabolite β-hydroxy-β-methylbutyrate (HMB) can increase muscle protein synthesis via mTORC1 activation^160^ alongside improved muscle function and reduced fibrosis^161,162^, which has been confirmed in humans^163^. Thus, administration of relevant dietary supplements to enhance mTORC1 immediately after trauma could be one avenue to suppress catabolic effects of trauma injuries, although more research is warranted given the surprising finding that disuse atrophy could mediate sustained mTORC1 activation that in turn disrupts autophagy homoeostasis to induce fibre atrophy via inhibition of upstream sestrin signalling^87^. Another key mechanism related to activation of mTORC1 is also the mechanism of mechanosensing, with increased mechanical loads known to activate mTORC1 signalling and protein synthesis by tension-linked membrane signalling (i.e., via phosphatidic acid)^159^. As such, early activation of mechanosensitive molecules could be another avenue worthy of target, particularly as loss of mechanosensor integrity in muscle fibres is known to release sarcomere-bound transcription factors related to the atrogene programme^95^, while upholding of the z-disk anchored structural protein titin is also known to be vital for sarcomere maintainence^164^ and linked to control of muscle mass^165^ (Fig. 5). With ballistic wounds, surgical debridement to treat firearm wounds (i.e., excision of injured tissue) is often associated with removal of healthy tissue and success is highly variable, which can impact muscle recovery. Recent preclinical studies using topical bromelain treatment^166^, which has selective proteolytic properties, demonstrated improved recovery of injured muscle in the hindlimb pigs subjected to gunshot wounds when used alongside simple wound incision over surgical debridement. Overall encouraging drugs have recently emerged that have anti-catabolic and pro-anabolic effects^81^, however, many of these currently require rapid translation to humans to confirm efficacy.

### Angiogenic therapies

Throughout this review, we have highlighted how vascular homoeostasis is essential for muscle regeneration and maintenance of muscle mass. Angiogenic growth factors with some specificity have already been used in clinical trials to stimulate new blood vessel growth and restore flow to ischaemic heart, limbs, and brain; and to heal wounds^102,167,168^. Angiogenic gene therapy has been trialled as a method to deliver angiogenic growth factors, with limited success, and current attention is largely focussed on programmed cell therapy (Fig. 5). The list of factors that are known to stimulate or inhibit angiogenesis is extensive—some offer common responses to a variety of stimuli, others are stimulus-specific, and in some cases the same factor (such as ephrins) may have dual roles depending on stimuli eliciting the response—but the key player is VEGF, present in many isoforms and acting through a range of receptor tyrosine kinases. Therefore, an attractive option is to use cell-based therapy as a mechanism of paracrine signalling, allowing intrinsic feedback mechanisms to direct both the titre and mix of releasate. Identifying the best candidate has proven difficult: only 50% of proliferating cells at the site of angiogenic capillaries are EC, the rest are fibroblasts; mast cells are not important in physiological angiogenesis but degranulation is important in inflammatory angiogenesis; greater numbers of ED1- and ED2 + ve macrophages are co-localised to VEGF + ve capillaries after 2 days of stimulation^106^; TIE2-expressing monocytes/macrophages regulate revascularisation of the ischaemic limb^169^. However, the role of stromal cells depends on the angiogenic stimulus: perivascular pericytes may regress with muscle stimulation, maintain a constant ratio with EC with hyperaemia, and increase coverage during overload^170^.

There are many possible drivers of angiogenesis in addition to chemical stimulation; increased shear stress, chronic muscle stretch and passive exercise offer similarly potential angiotherapies that may prove useful in muscle regeneration^171^ (Fig. 5). However, when considering the effect of rehabilitation strategies it is important to be cautious of quick fixes, as re-establishing haemodynamic control and effective nutrient exchange likely requires a return to a vascular network that is close to the original organisation and phenotype. For example, following recovery from crush damage, muscle fibre-type grouping may accompany reinnervation, thereby influencing recruitment pattern or fine motor control. Apparent early success with gene therapy (using VEGF, FGF-1 or HGF plasmids) gave transient relief from chronic limb ischaemia in those patients who were not candidates for conventional revascularisation procedures, but with limited success in avoiding amputation and variable improvement in other clinical outcomes (a plethora of collateral vessels does not guarantee success)^172^. Failure to elicit adequate transformation, even after 1 year of indirect electrical stimulation^173^, left muscle inadequate for transplant purposes^174^, which along with development of mechanical alternatives, led skeletal muscle as an effective cardiac assist device to be abandoned. These observations are consistent with the concept that angiogenesis requires overlapping, integrative responses. Chemical signals may dominate in pathological angiogenesis (tumours, inflammation, wound healing), often requiring a combination of factors to elicit an effective response, whereas mechanical signals dominate in physiological remodelling (ontogenetic growth, training), but optimised rehabilitation may require a judicious combination^175^.

Finally, bioengineered angiogenic solutions are being explored. Traumatic skeletal muscle injuries cause irreversible tissue damage and impaired revascularisation, suggesting the use of ‘engineered’ muscle transplantation may be useful^176^. Currently, murine skeletal myoblasts have been co-cultured with EC in aligned nanofibrillar scaffolds to form endothelialised and aligned myotubes, showing synchronised contractility and abundant secretion of angiogenic cytokines^177^. Treatment of traumatically injured muscle with endothelialised and aligned skeletal muscle promotes the formation of highly organised myofibers and microvasculature, along with greater vascular perfusion, and may offer a therapeutic option when other avenues are not feasible. To complement these approaches, programmed cell therapy is being vigorously pursued. Here, stem cell-based regenerative therapy is proving to be a promising strategy for the treatment of severe muscle diseases. Pluripotent stem cells (e.g., human iPS) may be necessary for long-term survival of transplanted three-dimensional (3D) engineered tissues in vivo, in order to induce essential capillary growth into the engineered tissues after transplantation^178^. In addition, satellite cell activation and their ability to differentiate may be related to proximity to capillaries, likely reflecting diffusion limitations. Most satellite cells in mouse and human muscle are within 5 μm of a capillary, and active satellite cells are located closer than quiescent satellite cells^179^. Capillaries and satellite cells can reciprocally activate each other via diffusion of secreted growth factors (IGF, HGF, VEGF), such that a denser capillary network is associated with a larger SC activation and expansion^106^, and a more pronounced hypertrophic response to resistance exercise^180^. Thus, targeting the endothelium may be an alternative approach for enhancing satellite cells activation and muscle regeneration following ballistic trauma, and this should be considered a major organ to target early in the recovery process.

## Future directions

This review has discussed some key topics related to skeletal muscle damage and regeneration following firearm-related wounds. While it is encouraging to see many advances in this field that have helped improve our understanding and treatment regarding muscle-related trauma, there are clear gaps in knowledge and medical treatment that need to be addressed. After reviewing the current literature and clinical management of ballistic muscle injuries, we have identified a number of major issues we believe warrant further attention, including:

- Firearm laws and access: the evidence is clear that reducing access to firearms results in fewer injuries (both fatal and non-fatal). Thus, preventing firearm injuries could be achieved in many instances if access became more tightly regulated by governments across the world (with sales of firearms subjected to criminal background check, appropriate educational programme of firearms safety, mental health evaluation, etc)^181^. Implementing educational classes highlighting the devastating physical, social, and economical consequences of firearms should be more common, with easier accessibility to this from an early age as most gun-related injuries involve young males. Setting tighter responsibilities for firearm owners regarding safe storage can also reduce incidents, especially accidents and suicide attempts involving children and teenagers^182^.
- Improved understanding of the basic mechanisms involved in ballistic muscle trauma: including strengthening our knowledge of the underling molecular alterations is essential to identify targets for therapeutic manipulation. This requires development of improved translational experimental models that better mimic ballistic trauma. Further research with robust experimental design, and well-controlled investigations in patients is urgently required to identify those aspects directly impacted by firearm injuries (rather than inferring data from indirect ballistic trauma settings, which may not translate well). We are also in dire need of more clinical trials to confirm the efficacy of current treatments for muscle-related firearm injuries.
- Improved treatment strategies: by harnessing innate tissue regenerative capacity and exogenous methods to optimise functional recovery after injury, are feasible. To do this, we require innovative drug discovery programmes to be launched that will enable rapid delivery of drugs at the geographical location of injury capable of acutely improving muscle regeneration (i.e., in the *golden hour*). Based on available evidence, early interventions that can enhance satellite cell repair, inhibit proteolysis, and promote angiogenesis are key therapeutic targets. In particular, advances in the area of engineered bioconstructs to recover muscle mass have been encouraging^124,125^, and this could represent an important treatment avenue to rescue even the most severe firearm-related muscle injuries. More focus is also warranted on a multidisciplinary approach to augment muscle regeneration following firearm wounds. In the long-term, more evidence is required to optimise rehabilitation regimes, including the most effective combination of exercise training regimes, nutritional supplements, and drugs that will ultimately aid a rapid recovery.

## Conclusions

Firearm-related skeletal muscle damage and regeneration involves an intricate balance between a variety of complex factors, including myogenic stem cells, protein synthesis/degradation, and angiogenesis. This review has highlighted that further research is required to better understand the underlying pathophysiological processes responsible for ballistic muscle trauma and the regenerative process. However, there is currently some exciting therapeutic approaches under development, which, if translated to clinical practice, will likely yield faster and improved recovery of patients plagued by firearm and blast-related injuries.
